# Improving Entropy Estimates of Complex Network Topology for the Characterization of Coupling in Dynamical Systems

**DOI:** 10.3390/e20110891

**Published:** 2018-11-20

**Authors:** Teddy Craciunescu, Andrea Murari, Michela Gelfusa

**Affiliations:** 1 National Institute for Laser, Plasma and Radiation Physics, RO-077125 Magurele-Bucharest, Romania; 2 Consorzio RFX (CNR, ENEA, INFN, Universita’ di Padova, Acciaierie Venete SpA), 35127 Padova, Italy; 3 EUROfusion Consortium, JET, Culham Science Centre, Abingdon OX14 3DB, UK; 4 Department of Industrial Engineering, University of Rome Tor Vergata, 00133 Rome, Italy

**Keywords:** system coupling, cross-visibility graphs, image entropy, geodesic distance

## Abstract

A new measure for the characterization of interconnected dynamical systems coupling is proposed. The method is based on the representation of time series as weighted cross-visibility networks. The weights are introduced as the metric distance between connected nodes. The structure of the networks, depending on the coupling strength, is quantified via the entropy of the weighted adjacency matrix. The method has been tested on several coupled model systems with different individual properties. The results show that the proposed measure is able to distinguish the degree of coupling of the studied dynamical systems. The original use of the geodesic distance on Gaussian manifolds as a metric distance, which is able to take into account the noise inherently superimposed on the experimental data, provides significantly better results in the calculation of the entropy, improving the reliability of the coupling estimates. The application to the interaction between the El Niño Southern Oscillation (ENSO) and the Indian Ocean Dipole and to the influence of ENSO on influenza pandemic occurrence illustrates the potential of the method for real-life problems.

## 1. Introduction

The synchronization between systems connected through some form of coupling is a common phenomenon occurring in a wide variety of fields, like physics, engineering, biology, physiology, secure communication, environmental sciences, etc. Several types of synchronization have been identified during the last decades: complete synchronization, where the interaction between two identical systems is strong enough to lead to in step trajectories after a transient period [1]; generalized synchronization, which refers to completely different systems where the dynamic variables of one system (the response system) are determined by the other system (the drive system) [2]; phase synchronization, where the phase difference is asymptotically bounded while the amplitudes remain weakly correlated [3]; lag synchronization, which implies the existence of an asymptotic bound between the output of one system and the time-delayed output of a second one [4]; intermittent lag synchronization, which is equivalent to a lag synchronization interrupted by intervals of non-synchronous behavior; and almost synchronization [5], which implies the existence of an asymptotic finite difference between certain subsets of the variables in the two systems.

The first attempt to formulate a unified definition and a general formalism for synchronization has been proposed by Brown and Kocarev [6]. The systems X and Y are considered to be synchronized, with respect to the properties $g_{x}$ and $g_{y}$, if there is a time independent mapping $h:{\mathbb{R}}^{k}\times {\mathbb{R}}^{k}\to {\mathbb{R}}^{k}$, *t* such that $\left\|h\left(g_{x},\;g_{y}\right)\right\|$ approaches zero asymptotically when the time t goes to infinity. The choices of $g_{x}$, $g_{y}$, and h determine the type of synchronization. Boccaletti et al. [7] proposed a simplified approach, which is based on the notion of prediction and the use of a synchronization function such that a particular point in X is mapped, uniquely, to one point in Y. Therefore, synchronization means prediction of one system’s values from another. However, a unifying framework for the study of synchronization of coupled dynamical systems is still an open problem. 

From a practical point of view, measuring the degree of synchronization in coupled dynamical systems and the identification of possible causal relations is an important problem. A wide variety of methods has been proposed. A simple indicator of synchronization is based on the mutual information [8], which can be expressed by means of the Kullback–Leibler divergence (i.e., relative entropy [9] as the measure of the information gained by replacing the distribution $p_{X}\times p_{Y}$, corresponding to the independence between X and Y, with the joint probability distribution $p_{XY}$—where $p_{Z}\left(k\right)$ represents the probability of the k-th state in the Z time series. The transfer entropy [10,11] extends the concept of mutual information estimating the influence of the state of X on the transition probabilities in Y, taking into account the past history of Y. Therefore, the synchronization and the causal relationship of time series are investigated on the basis of predictability and information transfer. The synchronization likelihood [12] is closely related to the concept of generalized mutual information as introduced by Pawelzik et al. [13] and it is able to deal with non-stationary dynamics. A measure based on quantifying the correlation dimension [14] of the coupled system, in comparison with the constituent subsystems, was proposed in [15]. Various indices of phase synchronization were also proposed [16,17]. Hempel et al. [18] developed a permutation-based asymmetric association measure. The method is based on the observation that two interacting subsystems are monotonically increasing functions. Synchronization can be determined also by monitoring the quasi-simultaneous appearances of certain predefined events in the time series [19]. The definition of the events is dependent on the particular application of the method. Methods based on topological criteria have been first proposed for quantifying generalized synchronization [20]. In a more general approach [21], attempting to detect causality relations between time series, the cross-convergent maps method looks for the signature of X in Y by checking the correspondence between points in the attractor manifold built from Y and points in the *X* manifold, where the two manifolds are constructed from lagged coordinates of the time-series variables X and X, respectively. An analytical measure based on the time rate of information flowing from one series to the other was proposed in [22], with the advantage that the resulting formula involves only commonly used statistics. A detailed evaluation of several synchronization measures is reported in [23]. Most of these methods are based the assumption that a stronger coupling leads to a stronger synchronization. As noticed in [23], different systems may have different behaviors during their transition to synchronization and the Lyapunov exponent is not always strictly monotonic. However, this assumption remains a practical method to evaluate the effect of the coupling strength variations especially for real-life data.

In this paper, we are proposing a measure that is based on transforming the time series into graphs by means of the “cross-visibility networks” (CVN) method [24]. The quantification of the coupling is obtained by estimating the entropy of the network topology. To improve the reliability of the results, the uncertainties in the measurements are taken into account with the innovative approach of the geodesic distance on Gaussian manifolds (GD). With regard to the structure of the paper, the next section describes the proposed method. Section 3 is devoted to the evaluation of the method’s performances by means of numerical tests, showing the advantages of adopting the GD in controlled conditions. For the demonstration of the approach potential to handle real-life data, an example from the field of atmospheric physics is presented in Section 4. Conclusions and indications of further developments are the subject of the last section of the paper.

## 2. Geodesic Distance to Improve Visibility Graphs for the Analysis of Synchronization Experiments

The transformation of time series into graphs was introduced to allow the study of time series dynamics by mean of the organization of networks. Zhang et al. [25] initially proposed to divide the time series into disjoint cycles and to consider each cycle as a node in a graph. Then the network representation can be obtained by connecting cycles for which the phase space distance is less than a predefined value. By using this method, the noisy periodic signals are mapped into random networks, while chaotic time series lead to complex networks exhibiting small-world and scale-free features [26]. 

**Figure 1:**
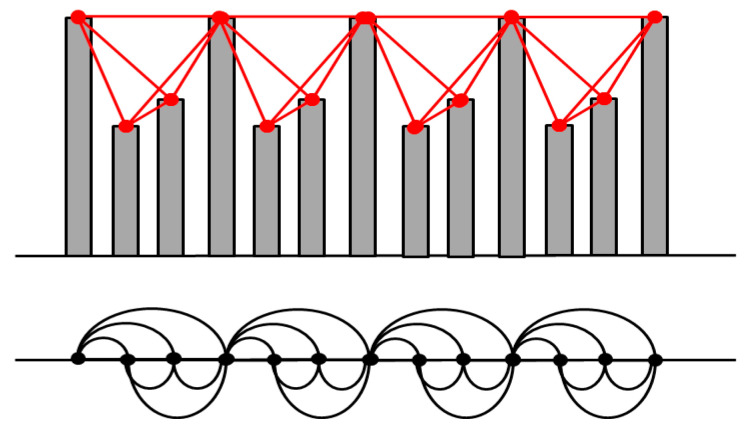
Illustration of the construction of a visibility graph from a time series.

In a very popular approach, Lacassa et al. [27] proposed to construct the mapping between the time domain and the network topology by considering a representation of time series using vertical bars; seeing this representation as a landscape, every bar in the time series is linked with those that can be seen from the top of the bar (Figure 1). Mathematically, two points, (*t_i_*, *y_i_*) and (*t_j_*, *y_j_*) in the time series, will be connected if the relation below is valid for any intermediate point (*t_k_*, *y_k_*):

(1) $$\;y_{k}<y_{j}+\left(y_{j}-y_{j}\right)\frac{t_{j}-t_{k}}{t_{j}-t_{i}}\;$$

The resulting complex network, called “visibility graph” (VG), is connected as each node can be linked at least with its first order neighbors in an undirected way. As proven in [27], the visibility graph inherits certain properties of the original time series. For example, a periodic series is converted into a regular graph, a random series into a random graph, and a fractal series into a scale-free graph. VG encapsulates the same amount of information as the initial time series but it may make more visible certain properties that are difficult to capture when directly analyzing the time series.

While the visibility graph represents a novel view for analyzing time series, the recently introduced cross-visibility networks have been explicitly conceived to reveal the possible coupling between them [24]. Considering a pair of time series $\left\{x_{i}\right\}$ and $\left\{y_{i}\right\}$, first they should be normalized (to their mean and standard deviation in case of stationary sequences and to maximum values for the non-stationary case) in order to make them comparable. Then the network is constructed by mapping each component of $\left\{x_{i}\right\}$ in a node of the graph. The connections are constructed by the following rules:

(2) $$\;y_{k}\leq y_{i}+\frac{x_{j}-x_{i}}{j-i}\left(k-i\right),\;i<\forall k<j\;$$

or

(3) $$\;y_{k}\geq y_{i}+\frac{x_{j}-x_{i}}{j-i}\left(k-i\right),\;i<\forall k<j$$

Therefore, the node i is looking at the components of $\left\{x_{i}\right\}$ time series, through the obstacles of the shifted time series $\left\{y_{k}\right\}$ = {$y_{k}$ − $y_{i}$ + $x_{i}$}. Equation (2) accounts for the visibility from the top view, while Equation (3) accounts for the visibility from the beneath view. Basically, the top view is determined by the reciprocal visibility of the peak values of the time series, whereas the beneath view by the reciprocal visibility of the valleys.

As emphasized in [24], the construction of the CVN is the result of local operations on the time series. This represents a different approach in comparison with methods like mutual information [8], Granger causality [28] and transfer entropy [10], which are investigating cross-correlations based on properties obtained by averaging over the whole times series. 

The constructed CVN can be represented by the adjacency matrix, whose elements are given by the relation:

(4) $$a_{ij}=\{\begin{array}{c} 1,\;if\;nodes\;i\;and\;j\;are\;connectd\\ 0,\;otherwise \end{array}\;$$

Several studies have shown that more robust results can be obtained from complex networks by weighting the graph connections (see, e.g., [29,30,31]). As an evolution of this approach, we have modified the network adjacency matrix by weighting the connections with the metric distance between two connected values in the time series:

(5) $$\;a_{ij}^{w}=\{\begin{array}{c} dist\left(y_{i}-y_{j}\right),\;if\;Equation\;\left(2\right)\;or\;Equation\;\left(3\right)is\;satified\\ 0,\;otherwise \end{array}$$

where $dist\left(y_{i}-y_{j}\right)$ is a distance. Using the Euclidean distance for this metric is a very popular choice but it implicitly assumes that the data points are infinitely precise values. However, this assumption is rarely satisfied in practical applications. In many cases, measurements are affected by various noise sources which can be considered, from the statistical point of view, as independent random variables. This will lead to measurements with a global Gaussian distribution around the most probable value, which is the value of the actual measured quantity. 

Therefore, a Gaussian probability density function, characterized by a specific mean μ and standard deviation σ, can be associated to each point in the time series data. In this view, the distance between two time series points is the distance between the corresponding Gaussian distributions, which can be calculated with the help of information geometry theory [32]. Various families of probability distribution functions (pdf) can be considered as lying on a Riemannian differential manifold. A point on this manifold corresponds to a specific pdf and the Fisher information constitutes a metric tensor (Fisher–Rao metric) on such manifold [33]. It can be demonstrated that the Fisher–Rao metric is unique, intrinsic and invariant under basic probabilistic transformations. For the case of two univariate Gaussian distributions $p_{1}\left(x|\mu_{1},\sigma_{1}\right)$ and $p_{2}\left(x|\mu_{2},\sigma_{2}\right)$, the geodesic distance (GD) on Gaussian manifolds is given by the relation:

(6) $$\;GD(p_{1}||p_{2})=\sqrt{2}ln\frac{1+\delta }{1-\delta }=2\sqrt{2}tanh^{-1}\delta \;$$

where:

$$\delta ={\left[\frac{{\left(\mu_{1}-\mu_{2}\right)}^{2}+2{\left(\sigma_{1}-\sigma_{2}\right)}^{2}}{{\left(\mu_{1}-\mu_{2}\right)}^{2}+2{\left(\sigma_{1}+\sigma_{2}\right)}^{2}}\right]}^{\frac{1}{2}}$$

**Figure 2:**
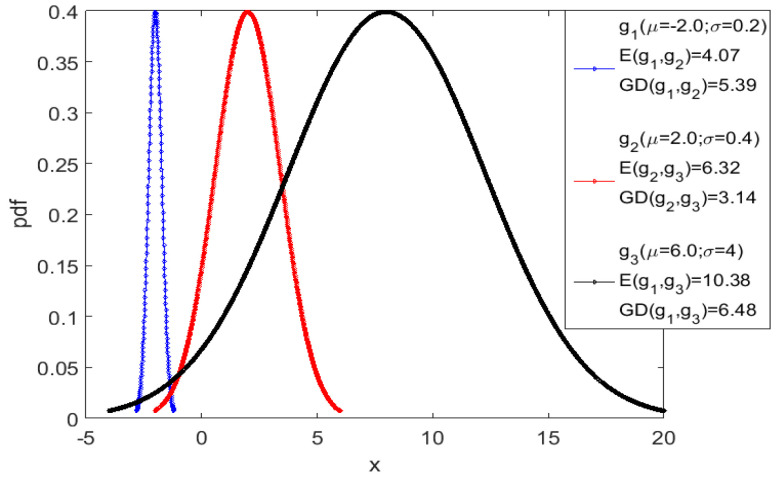
The geodesic distance GD between three Gaussian distributions $g_{1}\;\left(blue\right)$, $g_{2}$ (red), and $g_{3}$ (black) is compared to the Euclidean distance E between their modes. The Euclidean distance E(g1, g2) between g1 and g2 is less than half the distance E(g1, g3) between g1 and g3. On the other hand, since g3 has a standard deviation about an order of magnitudes higher than the other two, the geodesic distances GD(g1, g2) and GD(g1, g3) are almost the same [34].

An illustrative example, showing the difference between the Euclidean and geodesic distance, is presented in Figure 2. Further details regarding the use of GD for the analysis of noisy time series can be found in [34].

The weighted adjacency matrix (WAM) given by Equation (5) can be used to monitor the changes of the CVN structure when the coupling between the two-time series is varied. As the strength of the coupling increases, the complexity of the network decreases. An appropriate measure of the network complexity should be used in order to follow quantitatively this evolution.

The network complexity has been evaluated traditionally by means of measures like, e.g., degree distribution, clustering coefficient, edge density. More recently methods derived from information theory have been used. Many of them are based on the use of the Shannon entropy [35] and the underlying idea is to quantify the information content of the network as a measure of its “typicality”, as a tradeoff between random versus causal nature [36]. Implicitly, systems characterized by a low value of information, with low entropy, are considered to be “simple”. The network entropy is defined with respect to a network invariant. Most popular choices are the graph degree distribution [37] and adjacency matrices [38]. The last one offers more robustness against changes in the network size and is correlated with its algebraic properties [39]. Certain caveats should also be considered: the network entropy is based on the selection of a network invariant but it is not itself a network invariant [40]. It can be argued also that entropy, measuring statistical randomness, is not aligned with intuitive human understanding of complexity [41]. However, the entropy based measure is computationally affordable and a number of successful applications like, e.g., the analysis of DNA sequences [42] or in molecular biology [43] have been reported. 

The WAM adjacency matrix is expressed in real numbers instead of a binary representation and hence it can be represented as an image. Therefore, the image complexity evaluation is needed in this case in order to monitor the networks structure changes. Equivalently, image entropy will be used as a measure of complexity instead of network entropy. 

Image entropy can be formulated starting from the Shannon’s information theory [37] where entropy is used to measure the amount of information in a set of symbols such as an image. For a random variable X, the Shannon entropy is defined by:

(7) $$\;H\left(X\right)=-\underset{i}{\sum }P_{i}logP_{i}\;$$

where $P_{i}$ is the probability of the occurrence of $X=x_{i}$. 

The entropy for a grayscale image is equivalent to the above equation in which $p_{i}$ is calculated as follows:

(8) $$\;p_{i}=\frac{H\left(i\right)}{\sum_{i}H\left(i\right)},\;i=1,\ldots ,N_{H}\;$$

where H is the histogram of pixel intensities in the image, H(i) is the number of pixels with a certain intensity and $N_{H}$ the number of intensity bins in the image. 

A random noise image has the maximum value of entropy while the entropy of a uniform image equals zero. For usual images the areas characterized by a smooth changing of gray levels, the existence of blocks of uniform pixel values or the presence of repeating patterns of texture will have lower values than those for which the pixel values are changing rapidly or in a random way. Therefore, entropy reflects the non-uniformity and complexity of image texture [44,45].

The CVN structure evolves with the variation of the coupling strength. When the time series are not synchronized, the visibility of two points in the time series $\left\{y_{k}\right\}$ are more frequently interrupted by obstacles in the time series $\left\{x_{k}\right\}$. When the time series tends to synchronize due to increased coupling, the WAM evolves to a simpler structure, which translates into a relatively monotonic decrease of the image entropy (8). The entropy, as a measure of the degree of complexity, can therefore be used to define a measure of synchronization:

(9)  Q=−H(CVN) 

where the minus sign has been introduced in order to have an increase of Q with the coupling strength, coherent with most synchronization measures.

The synchronization measure should exhibit a monotonic behavior with the increase of coupling, to allow distinguishing the effects on synchronization. The assessment of a such behavior has been evaluated by using the degree of monotonicity defined in [23]:

(10) $$\;Mon\left(C\right)=\frac{2}{m\left(m-1\right)}\overset{m-1}{\underset{i=1}{\sum }}\overset{m}{\underset{j=i+1}{\sum }}sign\left(c_{i-}c_{j}\right)\;$$

where $C=\left\{c_{1},c_{2},\;\ldots ,\;c_{m}\right\}$ is the sequence of monotonically increasing coupling strengths, Mon(C)=1 for a strictly monotonical behavior.

## 3. Tests with Synthetic Data

The evaluation of the efficacy of the CVN entropy (ENT-CVN) measure in detecting different degrees of coupling has been tested first with synthetic data. We have considered three unidirectional coupled dynamical systems, based on strange attractors, that have been used also in [15,23]:

- the coupled Rössler system [46,47]:
  (11) $$\begin{array}{c} \;\dot{x_{2}}=0.95x_{1}+0.15x_{2}\\ \;\dot{x_{3}}=0.2+x_{3}\left(x_{1}-10\right)\\ \;\dot{y_{1}}=-1.05y_{2}-y_{3}+C\left(x_{1}-y_{1}\right)\\ \;\dot{y_{2}}=1.05y_{1}+0.15y_{2}\\ \;\dot{y_{3}}=0.2+\;y_{3}\left(y_{1}-10\right) \end{array}$$
- the coupled Hénon system [48,49]:
  (12) $$\begin{array}{c} \;x_{1}\left[n+1\right]=1.4-x_{1}^{2}\left[n\right]+0.3x_{2}\left[n\right]\\ \;x_{2}\left[n+1\right]=x_{1}\left[n\right]\\ \;y_{1}\left[n+1\right]=1.4-(Cx_{1}\left[n\right]y_{1}\left[n\right]+\left(1-C\right)y_{1}^{2}\left[n\right]\\ \;y_{2}\left[n+1\right]=y_{1}\left[n\right]\; \end{array}$$
- the coupled Lorenz system [50,51,52]:
  (13) $$\begin{array}{c} \;\dot{x_{1}}=10\left(x_{2}-x_{1}\right)\\ \;\dot{x_{2}}=x_{1}\left(28-x_{3}\right)-x_{2}\\ \;\dot{x_{3}}=x_{1}x_{2}-\frac{8}{3}x_{3}\\ \;\dot{y_{1}}=10\left(y_{2}-y_{1}\right)\\ \;\dot{y_{2}}=y_{1}\left(28.001-y_{3}\right)-y_{2}\\ \;\dot{y_{3}}=y_{1}y_{2}-\frac{8}{3}y_{3}+C\left(x_{3}-y_{3}\right)\; \end{array}$$

The time series $x_{2}$ and $y_{2}$ were used in case of Rössler and Hénon systems while the time series $x_{1}$ and $y_{1}$ were used in case of the Lorenz system.

For the Hénon system, the synchronization between the driver and the responder system is reached for C=0.8, as shown in [23], by following the plot of the responder attractor together with the plot of the driver versus responder components. For the Rössler and Lorenz systems, the evolution towards identity has been observed for C=2. Therefore, the coupling strength C has been varied in the interval [0,0.8], in steps of 0.01, for the Hénon system, while for the Rössler and Lorenz systems C evolves in the interval [0, 2], in steps of 0.2.

The analysis has been carried out using relatively short time series, with 4000 samples, typical of real-life applications. These applications are characterized also by the presence of significant levels of noise in the experimental measurements. As already mentioned above, we assumed that the measurements are typically affected by a wide range of noise sources, which are often independent and additive. Therefore, the uncertainties in the measured values can be considered Gaussian distributions. The theoretical models (11–13) have been used to generate first clean data. For testing the robustness of the proposed coupling measure against noise, realistic experimental conditions have been simulated by adding noise with an amplitude of 10% and 20% of the standard deviation of the original synthetic data. The mean value over 10 different noise realizations and 10 different initial conditions of the coupled systems has been considered.

**Figure 3:**
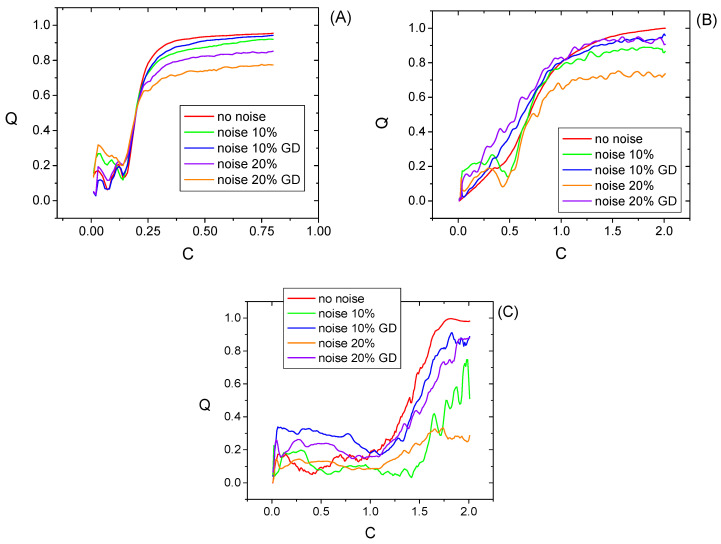
Dependence of the Q measure on the coupling strength for the Rössler (**A**), Hénon (**B**), and Lorenz (**C**). Q is normalized to its maximum value.

The evolution of the synchronization measure Q with the coupling strength C is presented in Figure 3, while the monotonicity values are listed in Table 1. In general, the evolution of the image entropy evolution is rather monotonic. An oscillatory behavior occurs in case of the Hénon system, but only for a very weak coupling C<0.15. For the Lorenz system the image entropy decreases have a slow rate and it affected by certain oscillations. This behavior is similar to that observed in [23] for other synchronization measures and it can be justified by the fluctuations of the maximum Lyapunov exponent of the responder system up to intermediate values of the coupling strength. The superimposed Gaussian noise leads to a deterioration of the monotonicity, in range of [8–14%] for 10% added noise and in the range of [13–23%] for 20% added noise. The use of the geodesic distance on Gaussian manifold in the WAM Equation (5) allows much better counteracting the effects of noise, providing systematically improved estimates. The monotonicity values are improved of 8–12% for the Hénon and Lorenz systems.

## 4. Real-World Applications

### 4.1. The Interaction between El Niño Southern Oscillation and the Indian Ocean Dipole

The identification of causal relations between time series has become an increasing focus of interest in climatology. In a pioneering approach reported in Marwan et al. [53], the influence of the El Niño Southern Oscillation (ENSO) irregular cyclicities, on the high variability of rainfall and river discharge in the Northwestern Argentine Andes, has been investigated by mean of cross-recurrence plots (CRP) [54]. Probably the most popular approach for identifying the coupling between time series in this field is based on the Granger causality technique [28] which exploits predictability to determine causation. It has been applied for exploring the causality between various phenomena like, e.g., global average observed time series of carbon dioxide and temperature [55], changes in level of atmospheric CO_2_ and the El Niño–Southern Oscillation [56], ENSO and rainfall-sensitive vegetation regions in Indonesia [57], atmosphere-ocean coupled circulation patterns and the global temperature variations [58], climate–vegetation dynamics [59], ENSO oscillation, and Indian summer monsoon (ISM) [60]. The coupling between ENSO and ISM has been investigated also by using statistical correlation tools [61]. The same kind of tools have been used also for investigating the causal influence of ENSO on the tropical plants reproduction and resource acquisition strategies [62]. The above list is far from comprehensive and it is intended only to give an idea about the increasing interest in this topic.

In order to validate the method proposed in this paper we will address the causal influence between ENSO and the Indian Ocean Dipole (IOD). This problem has been previously studied, using a similar methodology, but a different causation measure in [22]. 

ENSO is the most important coupled ocean-atmosphere phenomenon with profound consequences on the global climate and the ocean ecosystem on inter-annual time scales. It is believed to influence various phenomena, such as floods in South America and droughts in Southeast Asia and Southern Africa [63]. It has also been linked sea surface temperature (SST) anomalies over other ocean basins via the “atmospheric bridge” [64]. A typical example is IOD, which is also an air-sea coupled mode, which determines the SST periodic oscillations [65,66]. IOD influences the climate of Australia and of the countries surrounding the Indian Ocean Basin, determining high rainfall variability in this region. The general view assumes that IOD has a self-generating mechanism determined by the internal atmosphere–ocean coupling (see, e.g., [67]). However, increasing evidences on the existence of a link between ENSO and IOD have been reported: the occurrence of both El Niño/ La Niña and positive/negative events are described in [68]. ENSO events are able to influence the duration of IOD events. A reverse feedback may be induced by IOD on ENSO [69]. The ENSO–IOD interlink increased since 1970 together with the enhancement of the Walker circulation [70,71].

Several indices are used to monitor ENSO variations, all of them relying on sea surface temperatures (SST) anomalies averaged across a given region. The Niño-*n* (*n* = 1, 2, 3, 3.4, 4) indices correspond to regions crossed by different ships’ tracks, which have enabled the historic records of ENSO. In this paper we used the Nino-4 index (5N–5S, 160E–150W) which captures SST anomalies in the central equatorial Pacific [72]. The IOD intensity is represented by mean of the Dipole Mode Index (DMI), which is the SST gradient between the western equatorial Indian Ocean (50E–70E and 10S–10N) and the south eastern equatorial Indian Ocean (90E–110E and 10S–0N) [73]. 

The existence of the linkage between ENSO and IOD is studied in this paper by analyzing the causal influence between the Nino-4 index and the Indian Ocean SST and also between the IOD index and the Pacific Ocean SST. The Nino-4 monthly index and the SST gridded data (latitude starting at −88.0 and increasing northward per 2 degrees up to +88.0 and longitude starting at 0.0 and increasing eastward per 2 degrees up to 358.0) are from the NOAA ESRL Physical Sciences Division [72], while monthly DMI series has been retrieved from the SST gradient by the Japan Agency for Marine-Earth Science and Technology (JAMSTEC) [73]. We used data in between years 1958 and 2010 because DMI data is available only since 1958 and also for a fair comparison with the results reported in [22].

**Figure 4:**
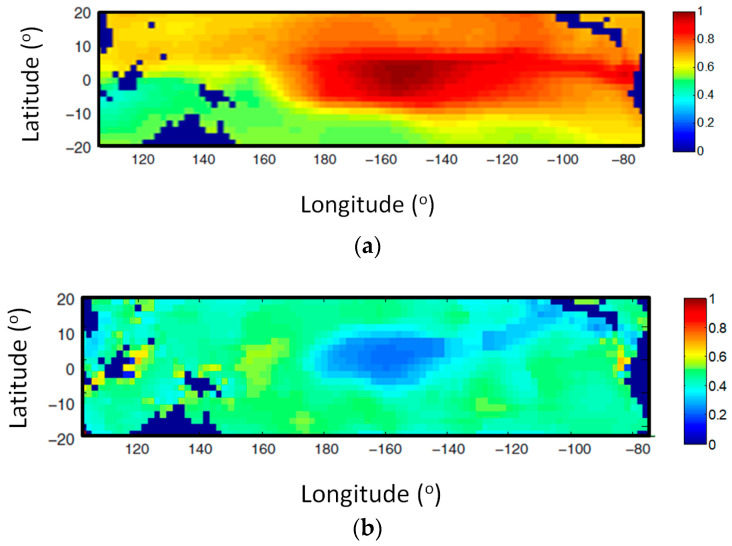
Map of the synchronization measure Q revealing the causation between the IOD index and the tropical Pacific SST (**a**), and vice versa (**b**). The Q map has been calculated with the spatial resolution of the SST gridded data (2 degrees for both latitude and longitude) for the time interval in between years 1958–2010.

**Figure 5:**
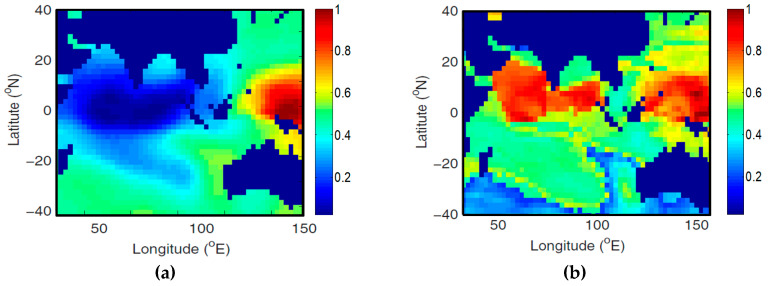
Map of the synchronization measure Q revealing the causation between Nino4 and the Indian Ocean SST (**a**) and vice-versa (**b**). The Q map has been calculated with the spatial resolution of the SST gridded data (2 degrees for both latitude and longitude) for the time interval in between years 1958–2010.

The causal influence between the IOD index and the tropical Pacific SST is presented in Figure 4 which shows a clear causal influence characterized by an El Nino-like pattern. Figure 5 shows the causation between Nino4 and the Indian Ocean SST. The causation map is characterized by two poles. The map of the feedback from the Indian Ocean SST (Figure 5a, shows two positive poles, revealing the influence of the Indian Ocean on El Nino by mean of IOD. This confirms the findings first reported in Figure 5b of [22]. This influence is extremely important as it may lead to the amplifications of El Nino oscillations.

### 4.2. The Influence of the El Nino Southern Oscillation on Influenza Pandemic Occurrence

The analysis of the causal relation between climatologic phenomena and the outbreak of various diseases by means of time series analysis represents a relatively new topic. For example, the effect of ENSO on the leptospirosis outbreaks in New Caledonia has been investigated in [74]. It has been found that La Niña periods are associated with high rainfall and both these factors are temporarily associated with outbreaks of leptospirosis. Moreover, it was possible to forecast the outbreaks for the next few months based on the sea surface temperature. This opens the possibility of an effective preparation of the health authorities. The ENSO-driven climate variability connection with periodic major outbreaks of dengue (a mosquito-borne viral disease) in Venezuela has been studied in [75]. The most significant dengue events correspond to the warmer and dryer years of El Niño. It has been shown that the ENSO variations, on seasonal and inter-annual scales, drive the occurrence of dengue periodicity through local changes in temperature and rainfall. The relationship between the dengue incidence in 14 island nations of the South Pacific and ENSO has also been investigated in [76]. Positive correlations have been investigated in 10 cases. Propagation between neighboring islands proved to be related only to modulating factors (such as population density and travel) and independent of inter-annual climate variations. In [77] it has been shown that the cholera dynamics in Bangladesh is characterized by an inter-annual component, which corresponds to the dominant frequency of ENSO. For a review of the present understanding of ENSO health associations the reader is referred to [78]. The review advocates the idea that, as ENSO is a complex non-canonical phenomenon, simple correlations are not able to correctly describe the linkage with different health phenomena and that the analysis should use tools more sophisticated than purely statistical ones. 

In this paper we analyze the causal relation between ENSO and the influenza pandemic occurrence. The incidence of the influenza epidemic is determined by the seasonal variation in virulence, transmission, and survival but also by the climatic factors. In particular ENSO induces the modulation the global precipitation (on time scales which extends from sub-annual to multi-decadal) [79]. A low precipitation rate increases the duration of suspension of aerosols in the air [80], leading to an increase influenza epidemic, as the aerosols represents the most effective mode of viruses’ transmission.

This problem has been investigated previously in [81] by analyzing the Joint recurrence plot (JRP) constructed with the SOI and SST time series. It has been found that JRP revealed periodic, quasi-periodic, and chaotic regimes, dominated by chaos–chaos transitions. All peaks of multiple waves of the influenza pandemics can be related to high divergence of SOI and SST trajectories.

**Figure 6:**
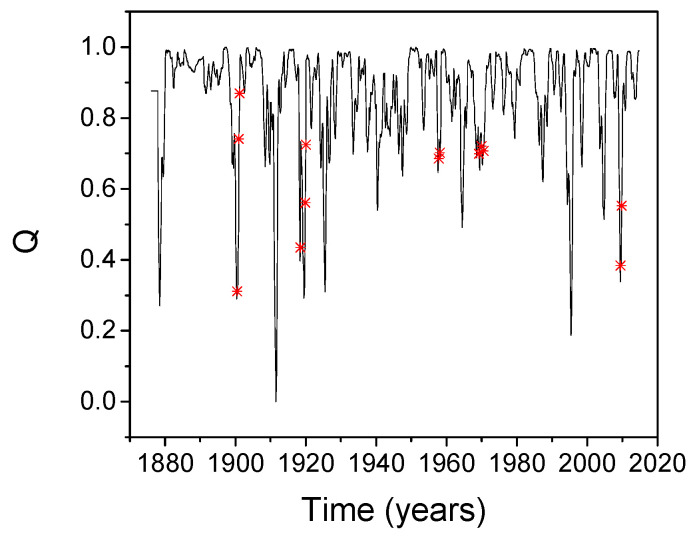
Synchronization measure Q calculated for the SOI and SST time series, using an 18-month sliding temporal window. The peaks of influenza pandemic waves are marked by red stars.

The problem has been analyzed in the present paper using a different tool. The coupling measure Q given by Equation (9) has been calculated for the SST and SOI time series, using a sliding temporal window of 18 months. Its evolution is presented in Figure 6 where the following historical records of onsets and peaks of influenza pandemic waves from 1876 to 2016 have been considered: December 1899, December 1900, March 1901, March 1918, July 1918, November 1919, January 1920, October 1957, February 1958, March 1969, December 1969, January 1970, June 2009, and October 2009 [81]. The pandemic waves coincide with a low correlation of the two processes. The results confirm, with a different and numerically independent method, the findings reported in [81].

## 5. Conclusions

The new method of weighted cross-visibility networks has been applied to time series for the investigation of the coupling between dynamical systems. The strength of the coupling is derived from the topology of the CVN and is quantified by the Shannon entropy of the adjacency matrix. The method introduces a new approach to time series causality evaluation. Additionally, the original use of the geodesic distance on Gaussian manifolds in the calculation of the entropy allows taking into account the errors in the measurements. The reliability of the coupling estimates is significantly improved in all the numerical cases investigated, for relatively low, but realistic, levels of noise up to 20%. The application of the technique to the interaction between ENSO and the Indian Ocean Dipole and also to the influence ENSO on influenza pandemic occurrence proves the potential of the approach to handle actual measurements for the investigation of complex systems. As the proposed methodology is able to provide robust results when working with short and noisy time series it is, therefore, very promising for the study of other complex systems, such as thermonuclear plasmas [82,83], in which different complicated phenomena, ranging from the physics of the fast ions and the impurities to the pacing of various instabilities, need to be better understood [84,85]. 

## Figures and Tables

**Table 1:** Evaluation of the monotonicity measure using clean data (CD), noise superimposed on data and Euclidian distance (N-ED) and noise superimposed on data and geodesic distance (N-GD).

	Rössler	Hénon	Lorenz
CD	0.92	0.95	0.89
10% noise, ED	0.84	0.83	0.75
10% noise, GD	0.88	0.91	0.83
20% noise, ED	0.79	0.74	0.71
20% noise, GD	0.81	0.86	0.82
